# Determining the critical factors of eWOM about corporate social responsibility on social networking sites: End users’ perspective

**DOI:** 10.3389/fpsyg.2022.894505

**Published:** 2022-09-08

**Authors:** Yuchen Hu, Qingbo Tang, Xuan Wang, Shahid Ali

**Affiliations:** ^1^School of Business Administration, Jiangxi University of Finance and Economics, Nanchang, China; ^2^Jiangxi Power Transmission and Transformation Engineering Co., Ltd, Nanchang, China; ^3^Department of Management Science, College of Management, Shenzhen University, Shenzhen, China; ^4^School of Economics and Management, North China Electric Power University, Beijing, China

**Keywords:** social networking sites, corporate social responsibility, electronic word of mouth, corporate image, structural equation modeling

## Abstract

It is now possible to propagate CSR information through social media platforms. Electronic word of mouth (eWOM) directly impacts image and upcoming portfolios of the organization. Customers, employees, and other stakeholders generate revenue for the company. Our goal was to understand why people were sharing and commenting in response to terrible reports about corporate social responsibility (CSR) on WeChat. A company’s desire to comment on and share CSR news and its perception of its own social and environmental responsibility were all presumed explanatory variables in our investigation. 315 WeChat users were asked to grade a fictitious news report of the environment. The results were shocking. According to our findings, an individual’s attitude toward actions and the effectiveness of information directly correlates to their social and environmental awareness level. EWOM may be discouraged by a company’s brand name, which has the potential to harm its reputation with its customers.

## Introduction

The management of stakeholder concern for responsible and irresponsible acts related to environmental, ethical, and social phenomena in a way that creates corporate benefit” is what CSR is all about (Zenggang et al., 2022). By linking “doing good” and “avoiding bad,” it allows companies to fulfill expectations of society and gain competitive advantages as a responsible performer while also helping them to “do good” over the long term (Diers-Lawson et al., 2020; Fernández et al., 2021). As a result of the demands of stakeholders, corporate social responsibility is essential (Sohaib et al., 2020; Yan et al., 2021; Wang and Majeed, 2022). When it comes to making purchasing decisions and building relationships with businesses, this issue is becoming more and more important to consumers (Abubakar et al., 2022). A new communication channel for disseminating CSR information has received little attention in prior research (Chen J. et al., 2021), primarily examining the effectiveness of communication channels (SNSs).

Companies and stakeholders can engage in a two-way conversation *via* social media, creating a new type of relationship (Johnstone and Lindh, 2021; Mar García-de Los Salmones et al., 2021). There are tens of hundreds of millions of consumers online, both individuals and businesses, who engage in a constant stream of conversation that has never been seen before (Khan et al., 2014; Ikram et al., 2019). Communication about CSR initiatives can be improved by using social networking sites (Chen X. et al., 2021). Social networking sites like WeChat, Twitter, and Instagram are “people’s media,” which means that users have the freedom to express both positive and negative feelings about organizations on these platforms. Alternatively, they could create a highly destructive form of publicity that could significantly affect its reputation and ability to attract new customers (Abdelmoety et al., 2022; Kumar et al., 2022). This is the topic of the present article.

According to the existing literature, customers evaluate and browse for electronic word-of-mouth (eWOM) for a product when making a purchase choice. It is, however, unclear how CSR communication on social media (CSRS) can lead to eWOM for a particular brand, based on an examination of the existing literature. However, most of these investigations are uncertain, even if they illustrate the significance of CSRS and eWOM (Liao et al., 2017; Martínez et al., 2021a). As a result, more investigation is needed in this area. As a result, the study’s primary goal is to implement the social identity theory to investigate how CSRS and eWOM are related (Williamson et al., 2006). According to the findings of this study, a third factor mediating the relationship between consumers and companies is called consumer–company identification (CCI).

A new form of electronic word of mouth (eWOM) has emerged that has the potential to have a considerable effect on a firm’s performance and prestige. Therefore, this study aims to determine the critical factors of eWOM about corporate social responsibility on social networking sites (Wechat). Internet-based applications that make it possible to create and exchange consumer content are referred to as “social media” (Sendlhofer, 2020; Achi et al., 2022). Blogs, social networking sites, content communities, online forums, message boards, and content creators are all included in this taxonomy proposed by Jiang and Park (2021). Since SNSs can easily spread information to a large audience, they have become the most successful of the five categories. This influences electronic word-of-mouth (eWOM) (Hai Ming et al., 2022). This paper offers three new ideas and perspectives to business and academic research.

First, CSR information is widely disseminated through the media, which is perceived as a credible and non-corporate source of information. CSR can benefit from the support of the news media, but journalists will always report on irresponsible behavior. The media covers small and large instances of irresponsible behavior on a daily basis. SNS can punish socially irresponsible companies by strongly moralizing CSR messages, taking into account that consumers have become more responsive to ethical and sustainability issues and that CSR information has a high impact on consumer engagement. According to our knowledge, no CSR-specific studies have been conducted on the relationship between news dissemination and social media to our knowledge. Furthermore, there is still a lack of research into the factors that compel people to share this type of news and produce eWOM.

Second, frequently cited aspect of corporate social responsibility (CSR) is the level of a company’s environmental performance. Companies’ environmental practices are becoming increasingly important because of the growing public concern for the environment (Sohaib et al., 2019). Consumers’ reactions to environmentally irresponsible behavior is rarely examined in the growing body of research on the influence of corporate environmental performance on their purchasing decisions (environmental degradation or pollution, among others). Nestle has been forced to take corrective measures by environmental activists because of its use of oil palm; Nike has also received unfavorable feedback about the labor practices of its suppliers, which has forced it to change its business practices. In this paper, we hope to shed some light on the factors that drive this type of behavior.

Finally, eWOM in social networks is measured as the intention to both comment and share on specific online content. Wechat have made it easier for people to go from passive consumers to active producers of content thanks to their support for user-created material. Replicating content (e.g., posting an existing newspaper article) and engaging in—but not creating—content are the primary goals of a “like” or “share” button. When a user leaves a comment on a website, they are able to engage in a conversation with the company. Due to the importance of CSR news, we decided to conduct an in-depth analysis of how people are reacting to the topic.

## Literature and research hypotheses

Stakeholders’ well-being and doing well have traditionally been considered the most important aspects of corporate social responsibility (CSR) (Silvestri and Veltri, 2020; Mattera et al., 2021; Castro et al., 2022). In addition, CSR is a term that means “avoiding bad” or “avoiding irresponsible acts” (Lau et al., 2016; Ahmad et al., 2021b). Environmental damage and unfair labor practices toward suppliers and employees are just a few long-standing complaints against corporations (Dey et al., 2018; Lin et al., 2020). The exposure of irresponsible behavior to the public can affect a variety of unfavorable outcomes, such as the loss of customers, a reduction in the motivation of employees, harm to the status, and negative electronic word of mouth (Huang et al., 2021).

CSR advantage of a company seems to have a specific influence on customers; studies show that consumers are more responsive to negative than positive CSR information and respond more intensely (Mar García-de Los Salmones et al., 2021; Hayes et al., 2022). According to the findings of various pieces of research, the way in which people process information in their heads before the occurrence of something negative (unfavorable or threatening) is very different from how they process information in reaction to something positive (beneficial or uplifting) (Martínez et al., 2021a; Hai Ming et al., 2022). As a result, when individuals revel in reckless manners, they will spend much more time considering it and looking for fundamental details; their decisions and reactions will be much more intensive (Chen et al., 2020; Fatma et al., 2020; Chukwuere et al., 2021; Lei et al., 2021; Deng and Zhao, 2022).

In consumer behavior research, one of the most widely held and broadly supported ideas is that electronic word-of-mouth communication has a significant impact on how consumers think and act (Gao et al., 2020). Customer dissatisfaction with goods, services, or businesses has long been regarded as an important avenue for eWOM communication.

### Environmental CSR awareness

There is a possibility that the personal qualities of the customer will affect their opinions and feelings in response to the stimulus (Ma et al., 2021; Castro et al., 2022). The effect of a person’s data may be different from one individual to another, and the same content may elicit various responses from various receivers (Ikram et al., 2019; Yang and Basile, 2021). As a result, the eWOM intention model may include environmental awareness and social awareness as important key factors. Numerous researchers have pointed out that word-of-mouth marketing has become increasingly important on social media (Mohamud, 2018; Kraus et al., 2020) (Ma et al., 2022). E-word-of-mouth on social networking sites can be conceptually broken down into three categories: opinion seeking, opinion giving, and opinion passing (Banks et al., 2014; Chaudhary and Akhouri, 2018). According to the findings of previous studies, both offline and online word-of-mouth rely heavily on opinion gaining and opinion granting as two of their most important defining characteristics (Ma and Zhu, 2022). However, this research focuses on the transmission of opinions because it is recognized as a neglected domain of electronic word-of-mouth on social networking sites and, as a result, requires careful research (Rasheed et al., 2018).

On the one hand, the term “social awareness” refers to a person’s consideration for society and the people living within it (Md Fudzee et al., 2017; Hens et al., 2018; Chen X. et al., 2021; Wu and Zhu, 2021). However, in contrast to this, environmental awareness refers to a set of specific psychological factors that are associated with an individual’s tendency to connect in pro-environmental behaviors and to make environmentally-friendly shopping behavior (Martínez-Ferrero et al., 2015; Kelley et al., 2019; Ahmad et al., 2021a). People who are socially aware of how others inspire them, as well as how their connections may influence others (Velasco Vizcaíno et al., 2021), are distinguished by their awareness and gratitude for the welfare of other people, as well as the enhancement of the standard of living and the advancement of financial development in their societies, and the preservation of natural resources (Rettab et al., 2009; Zhuang et al., 2021; Jang et al., 2022). Such people act in a manner consistent with their interests and concerns regarding societal issues, with the environmental aspect playing an especially significant role. (Liu et al., 2019; Horng and Wu, 2020; Martínez et al., 2021a).

According to Bhattacharyya and Bose (2020), the concepts of environmental and social parameters are distinct from one another, but they are connected (Matthes et al., 2020). According to Williams et al. (2020), people who give more weight to the importance of collective, or prosocial, values are more likely to be concerned about the environment and hold stronger pro-environmental beliefs (Moisescu et al., 2021). According to the theory put forth by Tajvidi et al. (2020), for individuals to engage in pro-environmental behavior, they need to stop thinking only about themselves and start thinking about the larger society as a whole. People’s prosaically tendencies would be evidenced in their interaction with the environment (Lee et al., 2016), leading to pro-environmental behaviors. This is because being prosocial requires people to benefit others, whereas engaging in these behaviors typically does not result in any direct personal benefits for the participant (Guo et al., 2020). We, therefore, propose that:

*H1*: Awareness of social and environmental issues has a positive effect on one another.

Communication about corporate social responsibility (CSR) will be more effective if it reaches people who are prosocial, CSR supporters, or activists; those people will be more likely to engage in the process, and the message will be more effective overall (Cao et al., 2021). Customers are more willing to devote the necessary amount of cognitive processing time to analyze the information content. Also, suppose they assume a message (such as an article about CSR) is relevant and significant. In that case, they will pay close attention to the quality of the arguments presented (for example, due to their social and environmental awareness). When people describe a message as relevant and important, they are more likely to view it as relevant and important. On the other hand, those individuals who believe that the same message has little personal relevance may not be willing to spend the time and effort necessary to evaluate it (Martínez et al., 2021b; Sohn and Kim, 2020). As a result, increasing the impact of a news story about potentially irresponsible corporate behavior can be achieved by raising awareness of environmental and social issues. We propose that:

*H2*: Social awareness has a positive impact on environmental corporate social responsibility (CSR) on We Chat.

*H3*: Environmental realization has positive impact on CSR information on We Chat.

### Information sharing

According to Rintanalert et al. (2021), the transition of worldviews has resulted in various levels of social awareness, ranging from people who are merely observers of the world around them (at the embedded level) to people who actively engage and have a powerful feeling of interconnection with other people. When a person is self-aware to the extent that their participation in the social environment has an effect. They feel motivated to take action, whether it be individual actions, the engaged group, and collaborative ones, such as experiences, sharing stories, and ideas. The various levels of awareness arise according to what the social environment can reflect, and awareness continues to expand so long as private expertise is publicly expressed (Basile and Vona, 2021). Because social networking sites are public media that enable users to communicate their thoughts and interests and build communities with one another, they can help this evolution along with the active participation of individuals concerning these issues (Barth et al., 2017).

*H4*: SNSs positively impact organizations’ environmental CSR content because of information sharing.

*H5*: Information exchange has a positive impact on commercial content on social networking sites (SNSs).

### Normative influence

Several studies have shown that electronic word-of-mouth marketing (eWOM) may be the most powerful source of information when consumers are susceptible to normative influence (Hooi et al., 2016). Consumer decision-making and the adoption of new technology are heavily influenced by normative influence (e.g., Carter and Easton, 2011; Zhang et al., 2015; Garcia et al., 2017). Normative influence can be divided into normative and informational (Ayoko et al., 2021). An individual’s behavior is affected by normative influences, which refer to a person’s inclination to conform to the social norms of others (Gerbens-Leenes et al., 2003; Donthu et al., 2021). In contrast, the tendency to accept information from knowledgeable people about a subject and to follow their lead when looking for a product, brand, or store is known as an informational influence (Derevianko, 2019).

Normative and informational influences can influence users’ eWOM behaviors in SNSs. Informational influencers in SNSs are more likely than others to seek advice and guidance from friends and acquaintances when making a purchase decision, which will aid in their participation in eWOM on SNSs. When it comes to purchasing and using products and brands that their significant others deem acceptable, consumers who are influenced by normative influences are more likely to adhere to these expectations.

To get their friends and family’s thoughts, they may turn to social media. As a result of the social influence of eWOM, SNS users see their contacts as valuable resources for product knowledge. Customers’ willingness to participate in eWOM on social networking sites (SNSs) can be explained by their receptivity to normative and informational influences. Hypotheses to investigate this phenomenon include the following:

*H6*: The normative influence of SNS users has positive impact on eWOM behaviors in SNSs.

*H7*: The informational influences of SNS users have positive impact on eWOM behaviors in SNSs.

### Corporate image

Social identity theory proposes that individuals feel a sense of belonging to a social group due to the emotional ties they share with members of that group. As a result, individuals identify themselves as members of the social group. In addition, according to the social identity theory, an organization can influence the behavior of its members by altering the members’ perceptions of themselves with the organization. This can bring about behavioral changes. Many different academics have extensively used the social identity theory to explain how consumers are encouraged to develop their identities and a sense of belonging with an organization engaged in CSR activities. Consumers are motivated to identify themselves with a socially responsible organization when an organization actively participates in corporate social responsibility (Zhang et al., 2019). As an illustration, Bolaños et al. (2022) developed a framework for CCI. They argued that CSR activities of an organization encourage consumers to identify with a particular organization because consumers anticipate that a socially responsible organization will satisfy their need for self-definition. This is why, when consumers’ self-concept is in line with various characteristics of CSR activities of an organization, they build a stronger identification with the organization, which stimulates them to evaluate a brand positively and enhances their positive behavioral intentions (Stanislavská et al., 2020).

*H8*: Share and commenting on negative news regarding CSR on SNSs are discouraged because of the company’s reputation.

### EWOM intention

Whether or not someone intends to like and share a WeChat comment is affected by their values, attitudes, and worldviews. People form an opinion about the usefulness of a post after reading it (Seopela and Zulu, 2022). Usability can be defined as an individual’s ability to use the information, as stated by (Kang et al., 2021). Because people motivated to process the information would take central routes, we predicted that this higher level of effort in cognitive processing would be more indicative of subsequent behavior than would be the case for people who took peripheral routes before this research (Vătămănescu et al., 2021). EWOM sources’ influence on user behavior has frequently been linked to user perceptions of the information available *via* these sources’ value (Mar García-de Los Salmones et al., 2021).

Dalla-Pria and Rodríguez-de-Dios (2022) found that eWOM adoption and opinion transmission positively correlate with this variable. Adopting or responding to a piece of information that is useful and interesting may be more likely when people are bombarded with so much information on social media (Khan et al., 2021). For example, studies have shown that content with high perceived interest or educational value (such as shock or surprise) is more likely to be shared than content that does not contain these elements. These factors have led us to the conclusion that:

*H9*: Useful information has a positive influence on eWOM behaviors in SNSs.

*H10*: A positive attitude toward sharing commercial content positively influences a positive attitude toward environmental CSR content.

*H11*: Sharing companies’ commercial content positively affects sharing companies’ environmental CSR content.

## Methodology

### Questionnaire development

In order to evaluate the concepts of the study, validated and accurate methods have evolved from the previous research in the field. These methods include the following: CSR dimensions from Fernández et al. (2021); effective commitment from (Fogel et al., 2018); and positive eWOM from Mattera et al. (2021). Every single construct accurately represents the corresponding concept. In order to collect the necessary information for the study, a standardized survey questionnaire was used. The demographic section can find the variables pertaining to gender, age, duration of stay, demographic origin, occupation, and educational level. The second section of this survey consists of questions designed to assess the level of affective commitment, positive e-WOM, and the three CSR activities. The responses were ranked using a Likert scale with seven points, one representing “strongly disagree” and seven representing “strongly agree” (Table 1).

**Table 1:** Measurement scale.

Dimension	Constructs	Explanation
Information Sharing	INT1	In order to distribute and discuss this information, I’ll primarily use social networking sites like We Chat
	INT2	Sharing and discussing this information with my friends is most likely to occur *via* social networking sites (SNSs)
	INT3	I went with social media to spread and discuss this piece of information
Normative influence	NORI1	I think this piece of news is worth reading
	NORI2	It’s my view that this piece of news is worth reading
	NORI3	I believe that the knowledge in this report is vital
Corporate image	CIMA1	I have a favorable impact on the NH company and am concerned it is a trustworthy source of information
	CIMA2	Using WeChat to discuss and share CSR news is, in my perception, a brilliant idea
	CIMA3	It’s a great idea to discuss and share CSR news on social media
Attitude and Beliefs	ATT1	CSR-related news should be distributed *via* social media, at least in my opinion
	ATT2	Being worried about the state of society makes me feel like a member of my neighborhood. I see myself as a person concerned about the welfare of others and the environment
	ATT3	I am concerned about my impact on the environment and strongly support environmental protection
Social awareness	SAW1	This data will be spread and commented on primarily through social networking sites (SNSs)
	SAW2	My companions are likely to hear about this news *via* social media
	SAW3	I went with social media to spread and discuss this piece of information
Environmental awareness	ENVC1	I think you should read this news item
	ENVC2	My opinion is that this article contains useful information
	ENVC3	As far as I’m concerned, this piece of news is essential

### Respondents’ selection and sample size

A large Southwestern university enlisted 400 undergraduates enrolled in elective advertising classes from across campus to participate in the research. Participants completed the study either for extra credit or to fulfill a class requirement. The final sample of 315 respondents was derived from the 400 participants who volunteered to participate in the study. Males comprised 49.5 percent of the sample, while females comprised 55.5 percent. People in the study were between the ages of 16 and 65. As a result, the population of SNS users represented by the sample was considered representative (Li and Ku, 2018a). Each major was described in the sample. Caucasians made up the bulk of the participants (58.1%), followed by Hispanics (14.3%), Asians (12.1%), and African Americans (4.1%).

### Statistical analysis

Statistical analyses are carried out using SPSS (edition 26) and AMOS (edition 26) software. The hypotheses are tested using structural equation modeling (SEM). In terms of practicality, SEM provides meaningful and accurate results, with three significant advantages over traditional methods (Li and Ku, 2018b). (1) An accurate evaluation of measurement errors. (2) Approximating the features of interest by using variables that have already been identified. (3) Modeling tool based on data compliance for trend evaluation and implementation (Lee et al., 2016). In addition, many multinomial strategies implicitly ignore math errors. However, the SEM makes predictions for both variables in the study while taking into account any errors in computation (Doha et al., 2019b). Due to the method’s dependability and serviceability, it produces accurate and scholarly numbers.

Predictor structures for each component may be generated using the SEM method, which also yields audio effects. Aside from that, it considers all of the mistakes made in work. As a result, the relationship between variables yields precise results (Al-Kubaisi and Abu-Shanab, 2022). Furthermore, a broad range of suppositions and complex interactions can be evaluated by integrating average arrangement and team market prices, which other designs and experiments may not be able to do (Song et al., 2021). This observation used SEM because that is the most productive technique for assessing the relationship between variables under analysis, both in and outside of analysis.

## Results and discussion

### Descriptive analysis, correlation analysis

Before beginning the actual research, the authors validated the questionnaire using a preliminary survey with a more limited participant pool to ensure that the findings would be reliable and informative (Cao et al., 2021). After that, we reached the participants by using a random sampling method to address that each and every individual of the subset carries an equal chance to be selected as a part of the data collection process to prevent unbiased representation of the population. This allowed us to ensure that we could avoid a situation in which the population was not accurately represented. This was done to ensure that the subset was representative of the population (Doha et al., 2019a). A method of sampling that is not based on probability was used to create the sample because the people who participated in the survey were all WeChat users. We provided respondents with a link to our online survey through Wechat. One month’s worth of time was allotted to the respondents so that they could complete the survey and give their responses. The respondents were given a comprehensive explanation of the questionnaire, including every one of its components. We were able to create quotas based on the percentages of users of WeChat who fell into each gender and age category. In order to meet the targets, we selected the responses at irregular intervals. Following the completion of processing and data collection, the total sample consists of 208 suitable surveys. The demographic information regarding the sample is provided in Table 2.

**Table 2:** Demographic of the sample.

	Percent (%)
**Gender**	
Male	45.455
Female	59.545
**Age**	
16–30	28.56
31–45	47.88
46–65	28.56

### Reliability analysis

The reliability of the measurement scales was evaluated with the help of Cronbach’s alpha, compound reliability, and AVE coefficients. Our results indicated that the measurement scales could be trusted to a certain extent. The notion that the constructs have a high degree of internal reliability is supported by the fact that the values of these statistics were, in every instance, higher than the required minimum values of 0.7 and 0.5, respectively (Li, 2019). In addition, the convergent validity of the scales was demonstrated by the fact that all of the items were discovered to be significant to a confidence level of at least 89%. Furthermore, they had higher-than-0.5 standardized λ coefficients (Osatuyi and Qin, 2018) (Tables 3 and 4).

**Table 3:** The data’s descriptive statistics.

Variables	Items	Observations	Coefficient of variation (CV)	Mean	Std. dev
ECSR	3	315	0.2602	4.5100	0.7118
CCSR	3	315	0.0449	3.3915	0.3773
EVA	4	315	0.1533	3.4734	0.5061
SAW	3	315	0.61005	2.709	1.5729
IFS	7	315	0.0819	3.24555	0.2541
NINF	7	315	0.1323	3.87135	0.48825
CIMG	5	315	0.2436	2.4885	0.5775
eWOM	4	315	0.28035	3.62565	0.96915

ECSR: Environmental CSR, CCSR: Commercial CSR, EVA: Environmental awareness, SAW: Social awareness, IFS: Information sharing, NINF: Normative influence, CIMG: Corporate image, eWOM: Electronic word of mouth.

**Table 4:** Confirmatory factor analysis (measurement model).

Variables	Items	Standard loadings	Cronbach-α	CR	AVE
Environmental awareness	EVA 1	0.753	0.878	0.843	0.530
	EVA 2	0.783			
	EVA 3	0.716			
	EVA 4	0.731			
Social awareness	SAW 1	0.876	0.963	0.984	0.715
	SAW 2	0.931			
	SAW 3	0.900			
	SAW 4	0.921			
	SAW 5	0.890			
	SAW 6	0.800			
	SAW 7	0.729			
Information sharing	IFS 1	0.790	0.959	0.983	0.710
	IFS 2	0.910			
	IFS 3	0.887			
	IFS 4	0.914			
	IFS 5	0.844			
	IFS 6	0.831			
	IFS 7	0.861			
Normative influence	NIFN 1	0.675	0.949	0.947	0.597
	NIFN 2	0.890			
	NIFN 3	0.813			
	NIFN 4	0.794			
	NIFN 5	0.813			
	NIFN 6	0.840			
	NIFN 7	0.696			
Environmental CSR content	ECSR 1	0.784	0.899	0.930	0.639
	ECSR 2	0.854			
	ECSR 3	0.826			
	ECSR 4	0.884			
	ECSR 5	0.742			
Commercial CSR	CCSR 1	0.799	0.752	0.815	0.751
	CCSR 2	0.915			
	CCSR 3	0.753			
Corporate image	OIM 1	0.659	0.802	0.879	0.600
	OIM 2	0.609			
	OIM 3	0.856			
	OIM 4	0.989			
eWOM	EWOM 1	0.791	0.858	0.868	0.571
	EWOM 2	0.731			
	EWOM 3	0.792			
	EWOM 4	0.783			

The results of the discriminate validity are presented in Table 5. Because the corresponding values for all constructs were significant and positive in this regard, it can be deduced that there is a strong correlation between each of the concepts.

**Table 5:** Discriminate validity analysis.

Variables	EVA	SOA	INS	NIFN	ENC	CIMG	eWOM
EVA	**0.74655**						
SAW	0.3423	**0.86625**					
IFS	0.28245	0.5124	**0.8631**				
NIFN	0.3696	0.3948	0.5512	**0.7917**			
ECSR	0.17535	0.5712	0.4368	0.32025	**0.8205**		
CIMG	0.32655	0.25095	0.189	0.33285	0.2331	**0.8785**	
eWOM	0.126	0.1323	0.10185	0.11655	0.0483	0.48615	**0.7927**

### Estimation of hypothesized structural model

**Figure 1:**
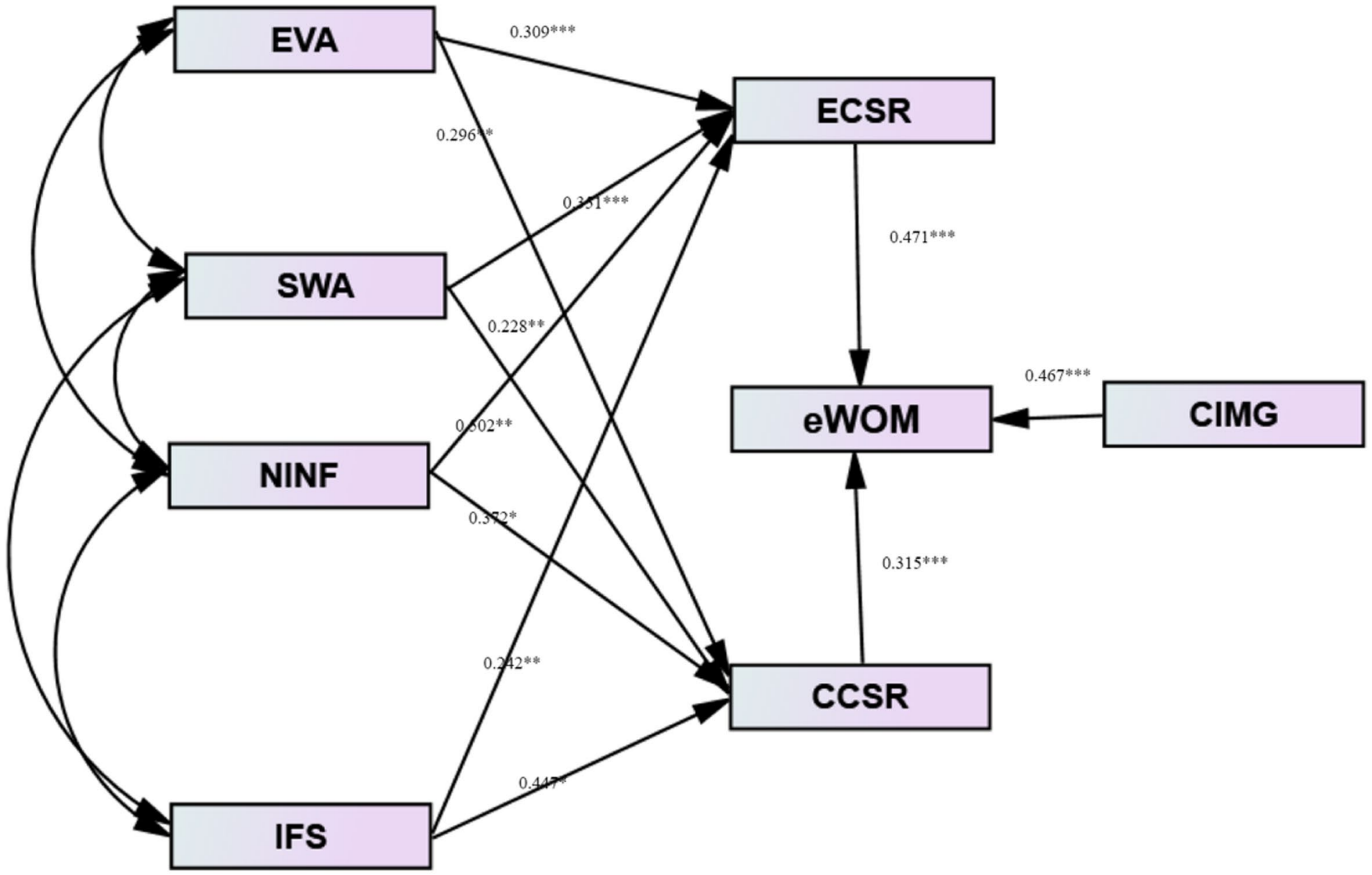
Path analysis.

After checking the predictive validity of the measures, the model’s validity was determined by applying the forceful maximum probability method Zoltan Balogh (2018). According to the findings of an initial investigation into the structural model, the level of environmental consciousness possessed by Customers’ perception does not have a massive effect on information’s relative benefits (hypothesis H3 is not supported). Consequently, we modified the research model and removed the non-significant affiliation, using the same methodology as Xiang et al. (2022), who developed the model structure approach. In Figure 1, the conclusion of the estimation of the recommended research models is displayed. These outcomes include the goodness-of-fit indices for the structural model.

The results (Normed 2 = 2.34; BBNFI 0.92), CFI (0.95) and IFi (0.95) showed that the structural model’s exact definition had been confirmed. RMSEA (0.08), on the other hand, was found to be inconsistent with the model (Table 6). H1: social awareness; H2: environmental awareness; and H3: attitude toward sharing and commenting, all influenced by empirical evidence (H4 is supported). WeChat users’ attitudes toward commenting and sharing on CSR news were affected by the environmental awareness (H4) but not by how useful they thought the information was (H3) (H5 is not supported).

**Table 6:** The outcomes of hypothesis testing on the structures.

Hypothesis	Structural paths	*β*-coefficient	*t-*Value	Decision
H1	EVA → ECSR	0.309^***^	2.447	Supported
H2	EVA → CCSR	0.296^**^	2.344	Supported
H3	SAW → ECSR	0.351^***^	3.294	Supported
H4	SAW → CCSR	0.228^**^	1.522	Supported
H5	NIFN → ECSR	0.502^**^	4.677	Supported
H6	NIFN → CCSR	0.372^*^	3.022	Supported
H7	IFS → ECSR	0.242^**^	1.597	Supported
H8	IFS → CCSR	0.447^*^	3.738	Supported
H9	ECSR → eWOM	0.471^***^	4.416	Supported
H10	CCSR → eWOM	0.315^***^	2.717	Supported
H11	CIMG → eWOM	0.467***	3.770	Supported

Both H6 and H7 have a direct bearing on whether or not the reader values the post’s content and whether or not they are willing to share and comment on WeChat (H6) (H8 is supported). Moreover, the corporate image adversely affected the aspire to respond and share to negative WeChat blogs about corporate social responsibility (H7 is supported). Consequently, negative eWOM about a corporate grows less quickly, at least in the beginning. User purposes of commenting and sharing on CSR blogs *via* WeChat are directly influenced by social and environmental awareness. However, other factors may affect it, such as their attitudes toward CSR and their perceptions of the benefits of CSR content providers.

### Discussion

People are becoming more concerned with issues of corporate social responsibility (CSR), and they are exposed to a variety of channels to receive information on this subject. The majority of communication occurs through unidirectional channels, such as advertisements, corporate social responsibility reports, or media coverage (Alraja and Hamdoun, 2021). However, the emergence of social networks as a new communication channel has brought about significant changes in the manner in which individuals and businesses exchange information and interact with one another. Users are able to become more integrated in the communication, creation, and exchange of content regarding a variety of topics through the use of social networks. This can include the users’ consumption experiences as well as other pertinent information that fits or reflects the users’ personal values (Balogh, 2018). With just the click of a mouse button, customers can either aid a business in its communication efforts or significantly damage its reputation and its prospects for future business (Sheng et al., 2022).

We came to the conclusion that social and environmental consciousness are key factors in explaining the intention to share and comment on a piece of negative CSR news. Because of the positive effect that it has on people’s perceptions of the usefulness of information and their attitudes toward sharing and commenting, social consciousness is one of the most important variables that the intention model takes into account. People who are socially conscious care about society and are committed to it; as a result, they pay attention to information that may have an impact on the community in which they live (Ikram et al., 2019). They will pay attention to a news story that contains information about the potentially irresponsible behavior of a company, and they will consider the information to be helpful, valuable, and important. The usefulness of the information has a direct bearing on the degree to which a person is inclined to comment and share a particular piece of news. Additionally, people who are prosocial have a favorable disposition to share and comment on CSR news on their social profiles, and this attitude toward behavior influences the intention that they have to spread the news.

Additionally, we discovered a strong correlation between social consciousness and environmental consciousness. We discovered that social consciousness leads to a greater awareness of the environment, and that both of these values influence attitudes regarding the sharing and commenting of CSR information on social networking sites. It comes as a surprise, however, that a significant correlation between environmental consciousness and the usefulness of information could not be established. One possible explanation lies in the manner in which the stimulus was presented. We created a post that was meant to be as realistic as possible by modeling it after an actual one. At first, we gave very little information, but it was sufficient to comprehend what was being discussed; however, we refrained from going into excessive detail (users would have to click to read the entire information). It is possible that people who are concerned about the environment need additional information in order to assess the magnitude of the event; this could explain why the event is not significant. Concern for anything that is related to the health and happiness of the community as a whole is an example of what is meant by the term “social consciousness,” which refers to a more general and less specific variable. Because the content of the post makes reference to a potential disruption to the established natural order, even though it is brief, it may be sufficient for users who priorities social responsibility to consider it valuable and helpful. It might be interesting to delve deeper into this matter by using different kinds of messages.

Last but not least, when people are made aware of a piece of news regarding a possibly irresponsible corporate behavior, which is not directly related to service failures but rather to issues concerning the environment, for example, the image of the corporation prevents them, at least initially, from discussing or sharing the news. People are aware that by engaging in this behavior, they are disseminating information that has the potential to be harmful to the company’s reputation; however, they believe that the corporate image will act as a shield in the interim, until they have more information regarding the event.

## Conclusion and policy implications

Companies are gradually becoming uncomfortable with objections to CSR variables, and they are exposed to a variety of outlets through which they can acquire knowledge on the subject. In addition, the number of outlets available to them is increasing. As a jumping-off point, the current research investigates the factors that contribute to the spread of negative eWOM among customers. By categorizing customers, recognizing what causes negative eWOM will be the primary goal of this study. According to this research, eWOM experience and e-mail communication priority directly affect negative eWOM intentions in both communities that participated in events and those that did not. This is an important finding because it sheds light on the relationship between these two factors. Specifically, previous experience with eWOM was shown to have a significant impact. This study indicates that unhappy customers who have previous experience with word-of-mouth marketing are more inclined to interact with negative word-of-mouth marketing intention. It is possible that this is because in the past when they voiced their negative emotions through online means, they experienced a sense of comfort that led them to anticipate having the same result in the future when they voice those emotions online. Customers who have prior experience with eWOM and are dissatisfied with the product or service they received may perceive that telling others about a negative utilization experience can help alleviate the negative feelings related to the experience (Caballero-Morales, 2021). As a result, people may be more likely to engage in negative eWOM if they feel this way. These findings were unanticipated but can be drawn from previous research (Pueyo, 2018). Additionally, customers who have a high level of online communication expertise are more likely to participate in expressing their displeasure actively. However, a dissatisfied customer who is new to eWOM and posting their thoughts about a bad shopping experience online may be unfamiliar with the online posting process.

### Implications for theory

The objective of the given paper is to identify the uniqueness that characterizes WeChat’s eWOM (purpose to comment and share). Whenever it relates to negative CSR data, like news about potentially irresolute environmentally friendly practices, eWOM, although previous research has focused on the negative effects of irresponsible manners, social media responses have been under-researched.

The results of this study, even though the study has some shortcomings, provide insights into the negative eWOM intentions that customers have regarding a product. One of its donations is to provide a framework that outlines how negative eWOM aims and antecedents interact. This serves as a starting point for the discussion.

### Managerial implications

In addition, the finding that participants who were exposed to the negative (as opposed to the positive) product review on the stranger’s blog site will be beneficial to advertisers who seek to utilize the potential of electronic word of mouth because it shows that the stranger’s readers are less likely to suggest the apartment to their associates (eWOM). Many business owners are offering monetary compensation to consumers who evaluate their brands on a personal blog (Huang, 2012). Incorporating eWOM branding through various SNSs is regarded as an essential component of the promotional mix. This research aimed to test a theoretical framework for analyzing the aspects of interpersonal relationships and how those aspects are related to eWOM in SNSs. It is important to note that the operationalization of eWOM in this research focused on product features and had implications for advertising. According to the findings of structural equations, trust, normative influence, and informational influence are positively associated with the overall eWOM behavior of SNS users on the site that they consider to be their favorite.

Public relations initiatives can be a useful tool for building relationships between companies and the media to develop strategies to avoid serious hazards to companies’ CSR performance. Companies that are serious about promoting environmental and social responsibility should cultivate their media relations and produce regular press releases to promote their CSR, gain publicity, and build a positive public image. Even trade shows, workshops, and press trips for journalists can be used to promote corporate social responsibility (CSR) in the same way as other public relations tools. Companies can also monitor and analyze social media conversations about CSR issues to learn more about how customers perceive a company or its CSR actions. As a result, businesses can gauge what their customers expect from them and incorporate that feedback into future CSR efforts. To avoid skepticism, companies should ensure that their CSR issues are presented and communicated truthfully before using public relations tools.

### Limitations and future research

One drawback of this research is that it only looked at one particular social networking site, such as WeChat. eWOM on CSR issues on other social networking sites could be the subject of future investigation (e.g., Twitter or Instagram). There may be valuable theoretical and managerial insights from comparing other social media (such as blogs). The results presented here could be generalized. SNSs for CSR reporting can be found in various places, including other online publications (such as journals, magazines, or broadcasts) and even business newspapers. Further research, given that this study focuses on the media’s role in disseminating that data. To broaden the scope of this discussion, since the researchers used a familiar and negative news point regarding environmental issues, it would be informative to introduce other assistance. On the other hand, this study has focused solely on the association connecting the factors of concern and has ignored other possible extraction and outcomes. As a result, additional mediating factors, such as people’s interest in CSR news, must be investigated.

Despite the study’s significant results, several limitations are to be considered. Using attribution theory, this study examined the impact of various online platforms on eWOM communication. To this day, it is not fairly obvious why users tend to attribute eWOM posted on personal blogs to circumstances, even though by analyzing causal attributions, this research presents a reasonable explanation for the impact of eWOM platforms on product evaluation. In the future, studies should look into why and how consumers choose to use specific eWOM platforms as a source of user-generated product information.

## Data availability statement

The raw data supporting the conclusions of this article will be made available by the authors, without undue reservation.

## Author contribution

All authors listed have made a substantial, direct, and intellectual contribution to the work and approved it for publication.

## Conflict of interest

QT was employed by Jiangxi Power Transmission and Transformation Engineering Co., Ltd.

The remaining authors declare that the research was conducted in the absence of any commercial or financial relationships that could be construed as a potential conflict of interest.

## Publisher’s note

All claims expressed in this article are solely those of the authors and do not necessarily represent those of their affiliated organizations, or those of the publisher, the editors and the reviewers. Any product that may be evaluated in this article, or claim that may be made by its manufacturer, is not guaranteed or endorsed by the publisher.
